# Liver transplantation for cryptogenic liver failure caused by diffuse hepatic angiosarcoma: case report

**DOI:** 10.1186/s40792-017-0296-0

**Published:** 2017-02-01

**Authors:** Yoshihiro Yoshida, Tomoharu Yoshizumi, Huanlin Wang, Kazuhito Sakata, Masahiro Shimokawa, Takeshi Kurihara, Takashi Motomura, Shinji Itoh, Noboru Harada, Norifumi Harimoto, Toru Ikegami, Hideaki Uchiyama, Yuji Soejima, Yoshihiko Maehara

**Affiliations:** Department of Surgery and Science, Graduate School of Medical Sciences, Kyushu University, Fukuoka, 812-8582 Japan

**Keywords:** Diffuse hepatic angiosarcoma, Living donor liver transplantation, Deceased donor liver transplantation, Cryptogenic liver failure

## Abstract

**Background:**

Primary hepatic angiosarcoma is a non-epithelial malignancy derived from sinusoidal endothelial cells, accounting for approximately 1.8% of primary hepatic malignancies. Diagnosis of primary hepatic angiosarcoma is complicated by difficulties in the qualitative radiological assessment of these tumors. Prognosis is very poor due to local recurrence and distant metastasis after liver resection or liver transplantation (LT).

**Case presentation:**

This case report describes two patients with primary hepatic angiosarcoma who were diagnosed by histopathological examination of the explanted liver after LT. One patient had undergone living donor LT, and the other had undergone deceased donor LT. Neither showed evidence of malignancy on the pre-operative imaging tests.

**Conclusions:**

Hepatic angiosarcoma has a very high relapse rate after LT. Pre-transplant liver biopsy may be necessary to distinguish diffuse hepatic angiosarcoma from tumors of other origin in patients with cryptogenic liver failure.

## Background

Cryptogenic liver failure remains an important cause of liver transplantation (LT) despite technical advances in imaging modalities and laboratory examinations [1, 2]. Hepatic angiosarcoma is a rare and aggressive malignancy with a poor prognosis and is difficult to diagnose in the absence of exposure to environmental carcinogens, such as thorotrast, polyvinyl chloride monomer, and arsenic [3, 4]. Diffuse type hepatic angiosarcoma is extremely rare and is very difficult to diagnose pre-operatively. Because these patients have a very poor prognosis after LT, owing to high rates of local recurrence and distant metastasis, LT is contraindicated in patients with these tumors [5]. As liver biopsy for patients with liver failure is risky, hepatic angiosarcoma is usually diagnosed by post-operative histopathological examination. This report describes two patients who underwent LT for cryptogenic liver failure and were found to have hepatic angiosarcoma on pathological examination of the explanted livers.

## Case presentation

### Patient 1

**Fig. 1:**
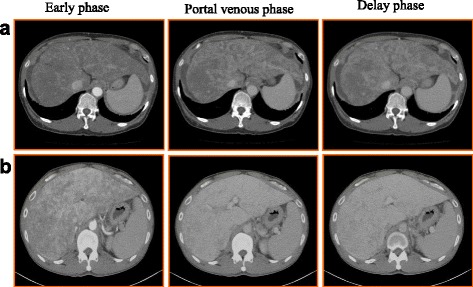
**a** Case 1. **b** Case 2. Computed tomography (CT) imaging revealed hepatosplenomegaly and the contrast effect of the liver was blocky. These findings may be an indicator of angiosarcoma

**Fig. 2:**
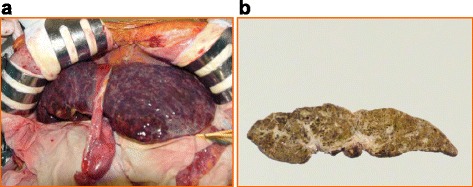
Recipient’s liver of case 1 **a** at laparotomy, and **b** macroscopic pathology showed *white spongy structure* in the explanted liver

**Fig. 3:**
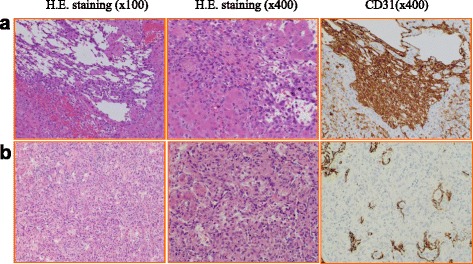
**a** Case 1. **b** Case 2. H.E. staining and immunohistochemical staining. Histopathological examinations showed dilated sinusoid lined by atypical spindle-shaped cells with polymorphic and hyperchromatic nuclei diffusely. These cells were CD31 positive with immunohistochemical staining

A 59-year-old female with esophageal varices and minor ascites visited a local hospital for abdominal pain. Blood tests revealed thrombocytopenia and mild liver dysfunction. She was negative for markers of viral hepatitis and autoimmunity, and her hepatic venous pressure was not elevated. Idiopathic portal hypertension was suspected. Ligation of endoscopic varices resulted in worsening of liver function. She was transferred to our hospital for living donor liver transplantation (LDLT). At admission, she was classified as Child-Pugh grade C with 11 points and her model for end-stage liver disease (MELD) score was 17. Abdominal contrast enhanced computed tomography (CT) revealed several small, low-intensity nodules in the liver but showed no definite tumors (Fig. 1a). Her 39-year-old son donated the left lobe of his liver for LDLT. Laparotomy revealed a soft and flabby liver, an enlarged spleen, and minor ascites. The post-LDLT course of the recipient was uneventful, and she was discharged from hospital 16 days after surgery. Histopathology of the explanted liver revealed spindle-shaped cells with nuclear variant and sinusoid expansion. The tumor was diffusely spread in the explanted liver and occupied about 20–30% of the liver (Fig. 2a, b). Immunohistochemically, these cells were positive for CD31, CD34, HHV-8, SMA, and ERG (Fig. 3a), resulting in a diagnosis of diffuse hepatic angiosarcoma. Contrast enhanced CT 9 months after LDLT showed recurrence of angiosarcoma in the transplanted liver. Despite systemic chemotherapy, she died 27 months after LDLT.

### Patient 2

A 43-year-old male visited a local hospital for abnormal liver function. Because his liver function was worsening, he was admitted to our hospital. Enhanced CT revealed hepatosplenomegaly. The contrast effect of the liver was blocky. No tumor in the liver was observed (Fig. 1b). He was classified as Child-Pugh grade C with 12 points, and his MELD score was 36. He underwent deceased donor liver transplantation (DDLT) from a brain dead 18-year-old male. His post-DDLT course was uneventful, and he was discharged from the hospital 22 days after surgery. Histopathology of the explanted liver revealed dilated sinusoid lined by atypical spindle-shaped cells with diffuse polymorphic and hyperchromatic nuclei. Immunohistochemically, these cells were positive for CD31 (Fig. 3b), but negative for CD34 and ERG. These features indicated diffuse hepatic angiosarcoma. His tumor recurred immediately after transplantation, and he died 6 months after DDLT.

## Discussion

About 2–3% of soft-tissue sarcomas in adults are angiosarcomas. Primary hepatic angiosarcoma is a rare malignancy, accounting for about 1.8% of all primary hepatic tumors and fewer than 5% of angiosarcomas [6, 7]. The peak age at onset is 60–70 years old, with a male-to-female predominance as high as 3:1. Although the main cause of hepatic angiosarcoma is exposure to environmental carcinogens, causes remain unknown in many patients [3, 4, 8]. Its symptoms are nonspecific and can include abdominal pain, weakness, fatigue, weight loss, anorexia, and abdominal enlargement. There is no relationship between hepatic angiosarcoma and portal hypertension. In the patient 1, however, the patient presented with esophageal varices. There are some case reports of hepatic angiosarcoma had associated with idiopathic portal hypertension, but the frequency is not high. The relationship is not clear. Diffuse hepatic angiosarcomas are especially difficult to diagnose by imaging modalities, although a blocky contrast effect of the whole liver in enhanced CT may be an indicator. These findings may suggest angiosarcomas, but they look very much alike hyperplastic nodules in liver cirrhosis. FDG-PET in one patient showed extensive diffuse abdominal accumulation throughout the whole liver, suggesting a malignant tumor [9, 10]. Histopathologically, these tumors are characterized by dilated sinusoids lined by hypertrophied endothelial cells with atypical and hyperchromatic nuclei. Immunohistochemistry has shown that these tumors are usually positive stains for endothelial markers, such as HHV-8, CD31, CD34, and ERG [10, 11]. About 7.7% of primary hepatic angiosarcomas are diffuse hepatic angiosarcomas [4]. Of 59,462 patients diagnosed with liver tumors from 1987 through 2003 in the UNOS database, seven (0.0117%) with diffuse hepatic angiosarcoma underwent liver transplantation and were pathologically diagnosed post-operatively. Their mean duration of survival after transplantation was 262 ± 146 days [12]. Owing to their poor prognosis and high recurrence rate, LT is contraindicated for patients with hepatic angiosarcomas, especially those with diffuse hepatic angiosarcomas [7]. In both cases, addition of chemotherapy such as IL-2 might be necessary after the established diagnoses.

## Conclusions

Liver biopsies are difficult to obtain from patients with cryptogenic liver failure. Nevertheless, pre-transplant liver biopsy may be necessary to distinguish diffuse hepatic angiosarcomas from tumors of other origin in patients with atypical cryptogenic liver failure.
